# Regrowth of zebrafish caudal fin regeneration is determined by the amputated length

**DOI:** 10.1038/s41598-020-57533-6

**Published:** 2020-01-20

**Authors:** Toshiaki Uemoto, Gembu Abe, Koji Tamura

**Affiliations:** Laboratory of Organ Morphogenesis, Department of Ecological Developmental Adaptability Life Sciences, Graduate School of Life Sciences, Tohoku University, Aobayama Aoba-ku, Sendai 980-8578 Japan

**Keywords:** Limb development, Regeneration

## Abstract

Fish have a high ability to regenerate fins, including the caudal fin. After caudal fin amputation, original bi-lobed morphology is reconstructed during its rapid regrowth. It is still controversial whether positional memory in the blastema cells regulates reconstruction of fin morphology as in amphibian limb regeneration, in which limb blastema cells located at the same proximal-distal level have the same positional identity. We investigated growth period and growth rate in zebrafish caudal fin regeneration. We found that both the growth period and growth rate differed for fin rays that were amputated at the same proximal-distal level, indicating that it takes different periods of time for fin rays to restore their original lengths after straight amputation. We also show that more proximal amputation takes longer period to reconstruct the original morphology/size than more distal amputation. Statistical analysis suggested that both the growth period/rate are determined by amputated length (depth) regardless of the fin ray identity along dorsal-ventral axis. In addition, we suggest the possibility that the structural/physical condition such as width of the fin ray at the amputation site (niche at the stump) may determine the growth period/rate.

## Introduction

**Figure 1:**
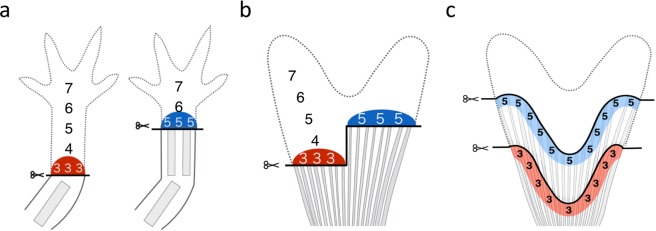
The concepts of positional identity and positional memory in regenerating limbs and fins. (**a**) Blastema cells in the limb memorize positional identity along the proximal-distal axis. Blastema cells in the limb located at the same proximal-distal level have the same positional identity. (**b**) A traditional concept suggests that blastema cells in the fin rays located at the same proximal-distal level have the same positional identity. (**c**) Our new model shows that niche at the amputated site located on a wavy line (the same distance from the tip of each fin ray) have the same positional identity.

Amphibians and fish have a higher ability than mammals to regenerate organs. They can regenerate organs that are seriously injured or lost, and their organ regeneration is often epimorphic. The process of epimorphic organ regeneration proceeds as follows: first, wound epidermis rapidly covers the surface of the amputated site, and then a blastema consisting of de-differentiated mesenchymal cells appears^1^. After blastema formation, appropriate tissues are newly formed from the blastema cells and reconstruct the original morphology. Studies on amphibian limb regeneration have indicated that the blastema cells may have positional memory^2–4^ (Fig. 1a). Limbs exhibit a pattern along the proximal-distal axis, such as the stylopod, zeugopod, and autopod. When the limb is amputated at a certain point on the proximal-distal axis, it must regenerate the tissues and morphological structure of the portion of the limb distal from the amputation site, according to so-called positional memory. Positional memory provides information about the relative position of each blastema cell in the limb (Fig. 1a, number 3 or 5 at the site of amputation) and about the structures that the blastema should reconstruct^2–6^. It has been suggested that positional memory also exists along the anterior-posterior and dorsal-ventral axes^7,8^.

Fish can completely regenerate an amputated caudal fin^9^. The caudal fin in zebrafish has a bi-lobed morphology, which can be divided into three regions: apparent dorsal and ventral lobe regions with longer fin rays and a cleft region in the middle with shorter fin rays (Supplementary Fig. S1)^10–13^. Here, we regard the position of the two lobes of the caudal fin as being along “the dorsal-ventral axis” in accordance with many reports on caudal fin regeneration^14–17^; however, to be exact, this axis is thought to be the posterior-anterior axis because the two lobes develop antero-posteriorly at the ventral side of the tail end and then bend up dorsally^18,19^. A fin ray consists of a proximal-distal series of two concave dermal bones (lepidotrichia) covered by epidermis, which bracket nerve tissues, blood vessels, and mesenchymal cells^20,21^. A regenerating fin forms a blastema, and cells of the blastema proliferate actively and re-differentiate to provide new fin rays^22^, similar to amphibian limb regeneration. Although de-differentiation, blastema formation, and tissue re-differentiation have been intensively studied in zebrafish, how the regenerating fin reconstructs its original morphology, that is, regeneration of the original length of the fin rays, remains unclear. It is unknown whether positional memory is also involved in zebrafish caudal fin regeneration to reconstruct the original morphology as with the amphibian limb regeneration (Fig. 1a,b). Proximally amputated fin rays grow faster than distally amputated ones^17,23^, suggesting that blastemas at different positions have different positional memory (expressed as growth rate). Furthermore, studies on the regeneration ability of transplanted fin rays demonstrated that positional memory in the blastema is involved in caudal fin regeneration^14,15^. On the other hand, other studies suggested that some conditions at the amputation site (niche at the stump) are rather important for restoration of fin length and reconstruction of fin morphology^24^. The contribution of positional memory in the blastema to the reconstruction of the whole morphology of the caudal fin is still under debate.

In this study, we investigated whether there are distinct characteristics of growth in each fin ray during caudal fin regeneration depending on fin ray identity along the dorsal-ventral axis and amputation level along proximal-distal axis, which would indicate that positional memory is used to reconstruct its morphology. First, we defined the termination point of reconstruction of fin morphology and measured the growth period and growth rate of each fin ray. By comparing the growth periods and rates of fin rays, we found that both the growth periods and rates are not the same for fin rays that are amputated at the same proximal-distal level. We also found that proximally amputated fin rays showed a longer growth period and higher growth rate. Statistical analysis showed that the values of the two parameters were correlated with the amputated length of the fin ray, regardless of fin ray identity, suggesting that fin rays amputated not at the same proximal-distal level (Fig. 1b) but rather on a wavy line (with the same length/depth from the distal tip; Fig. 1c) along the dorsal-ventral axis have same growth periods/rates. This does not correspond to the concept of positional memory in the blastema for amphibian limb regeneration. We will also show some evidence that the structural/physical condition (niche) at the amputation site (such as the width of fin rays) may determine the length that the amputated fin ray regenerates.

## Results

### Difference in growth period and rate among fin rays

In general, the final size of a growing animal structure such as an organ is expressed by two parameters: growth period and rate^25^. To define these parameters, the termination of regenerative growth must be recognized, but little is known about how long it takes to reconstruct the original morphology in fish fin regeneration. Previous studies mainly focused on a relatively early phase of fin regeneration, and the termination point of fin morphology reconstruction was not clearly defined. Thus, it is unknown how growth period and rate contribute to the reconstruction of the bi-lobed morphology. To track rapid fin growth and determine the termination point (day) of rapid growth, we used the ratio of fin length to body length (fin-to-body ratio; F-B ratio, see Method and Supplementary Fig. S2). Adult zebrafish maintain a certain F-B ratio as they grow isometrically throughout their life^26^. On the other hand, during the fin reconstruction process, the F-B ratio in each fin ray increases and eventually becomes constant when it approaches the pre-amputation value^27^ (Supplementary Fig. S3a). Thus, we defined the termination of rapid growth as the time point that a fin ray reaches constant growth (see Supplementary Method and Supplementary Figs. S3–S5).

**Figure 2:**
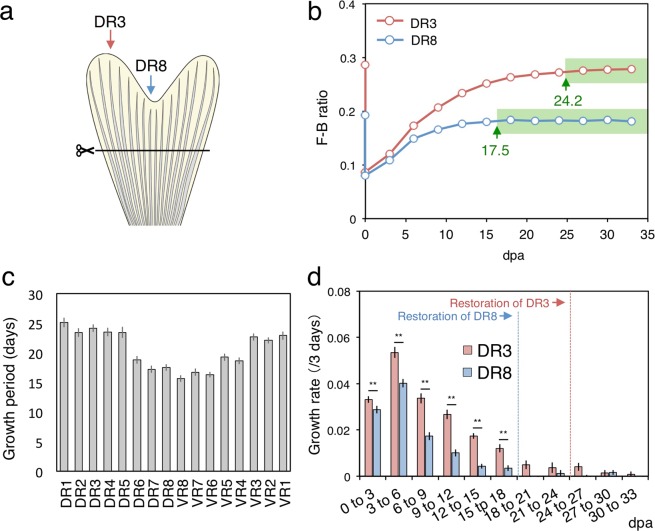
Growth analysis after straight amputation. (**a**) Schematic drawing of straight amputation. Fins were amputated with a straight cut along the dorsal-ventral axis. (**b**) Transition of the F-B ratio in DR3 and DR8. Green arrows indicate termination points. (**c**) Growth period of all fin rays. (**d**) Transition of the growth rate from 0 to 33 days post-amputation (dpa) in DR3 and DR8. Paired t-test: p-value ** < 0.01. Error bars means ± SEM.

To obtain and compare the growth period and rate among fin rays during caudal fin regeneration, we amputated the caudal fin (n = 19) parallel to the dorsal-ventral axis (straight amputation, Fig. 2a) at the position of the apex of a procurrent ray that was connected to the uroneural (Supplementary Fig. S1b, dotted line). We tracked the F-B ratio of all of the fin rays (DR1-DR8 and VR1-VR8; see Supplementary Method and Supplementary Fig. S1) every three days for 33 total days in the amputated caudal fins, calculated the growth rate, and evaluated the termination point of the restoration of fin ray length (arrows in Supplementary Fig. S6). For example, Fig. 2b shows the data for DR3 and DR8, in which the termination time point was determined to be 24.2 days post-amputation (dpa) and 17.5 dpa, meaning that the growth period was 24.2 days and 17.5 days long, respectively. These results indicated that there was 1 week difference in the growth period between these fin rays. We further found that the different growth period among fin rays depended on the fin ray length of before-amputation (Fig. 2c). Longer fin rays in the lobe regions exhibited a longer growth period than that of the shorter fin rays in the cleft region. Furthermore, the fin rays with the same length that were symmetrically positioned in the dorsal and ventral lobes showed similar growth periods. In addition, we compared the growth period of large and small fish. Although the actual fin size varied depending on fish size, fish in any sizes showed similar fin growth periods (Supplementary Fig. S7), suggesting that growth period in fin regeneration is independent of body and fin size.

Regarding the change in growth rate during fin ray regeneration, we found that for all fin rays, the growth rate was fastest at 3 to 6 dpa, and then gradually decreased (Fig. 2d, Supplementary Fig. S8). Throughout the growth period, the growth rate was always faster in DR3 (the longest fin ray) than in DR8 (the shortest fin ray) (Fig. 2d), suggesting that longer fin rays always regrow faster than shorter ones. In addition, the fin rays positioned symmetrically in the two lobes such as DR3 and VR3 showed a similar growth rate during the growth period (Supplementary Fig. S8).

**Figure 3:**
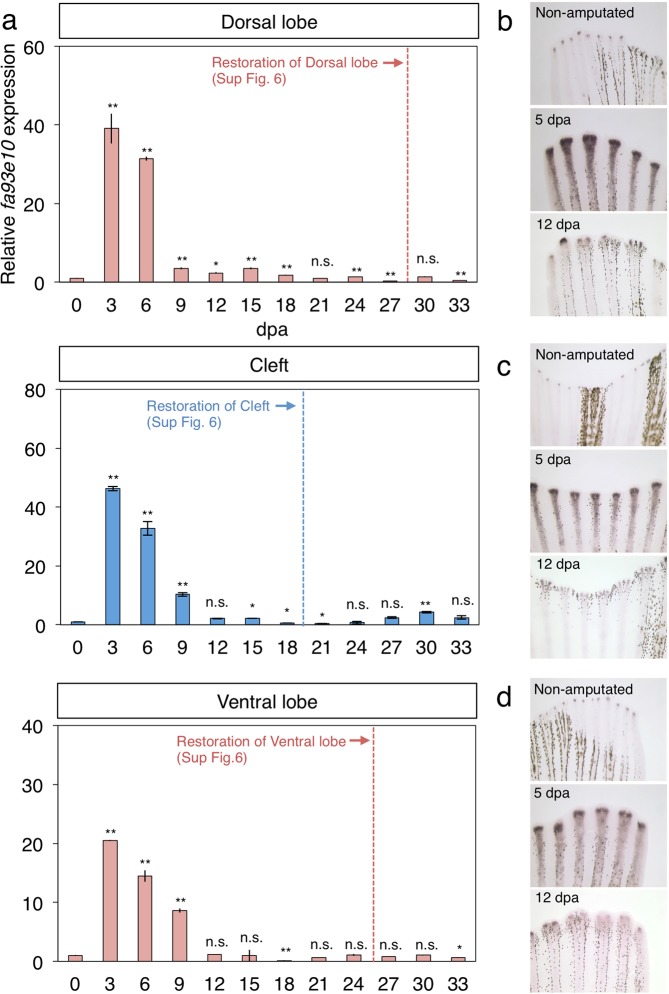
*fa93e10* expression analysis of regenerating fins. (**a**) qRT-PCR results for the Dorsal lobe (DR3 to DR5), Cleft region (DR7 and DR8, VR7 and VR8), and Ventral lobe (VR3 to VR5). Each time point n = 3. (**b**–**d**) *In situ* hybridization results of *fa93e10*. (**b**) Dorsal lobe. (**c**) Cleft region. (**d**) Ventral lobe. Welch’s t-test (compared 0 dpa with other time points): *p-value < 0.05, **p-value < 0.01. Error bars indicate ± standard error of measurement.

In the caudal fin, epidermal cells overlying proliferative distal mesenchymal cells express the *fa93e10* gene, which is known as a fin growth marker^27–29^. Quantitative reverse transcription polymerase chain reaction (qRT-PCR) for *fa93e10* (Fig. 3a) indicated that in both lobe and cleft regions, the expression level of *fa93e10* reached a peak at 3 dpa. This coincides with the peak of growth rate during fin regeneration (at 3 to 6 dpa, Fig. 2d and Supplementary Fig. S8), suggesting that cell proliferation contributed to growth rate. On the other hand, the expression level of *fa93e10* decreased drastically at 9 dpa and then became the same level as the pre-amputation at 21 dpa in dorsal lobe region, and decreased to the same level as the pre-amputation at 12 dpa in cleft region and ventral lobe region. Actual *fa93e10* expression in whole-mount *in situ* hybridization results also supported the qRT-PCR results (Fig. 3b–d and Supplementary Fig. S9). Attenuation of *fa93e10* expression was much earlier than the day of the termination time point (24.2 days, 17.5, and 22.7 days for DR3, DR8, and VR3, respectively), indicating that the time point of the attenuation of *fa93e10* expression did not coincide with the termination point of the restoration of the fin ray. Other factors such as matrix accumulation and cell shape change, as well as cell proliferation, may contribute to the later stage of rapid growth for morphological reconstruction.

### Differences in growth period and rate depend on the depth of amputation

Previous studies on zebrafish caudal fin regeneration postulated that it takes the same amount of time to restore the length of amputated fin rays regardless of the amputation depth, because proximally amputated fin rays regrow faster than distally amputated ones^17,23^. In addition, our results revealed that the growth period was different between the lobe (longer fin rays) and cleft (shorter fin rays) regions (Fig. 2c). Thus, we also postulated that each fin ray has a fixed growth period depending on the fin ray identity along the dorsal-ventral axis (DR1-8, VR1-8).

**Figure 4:**
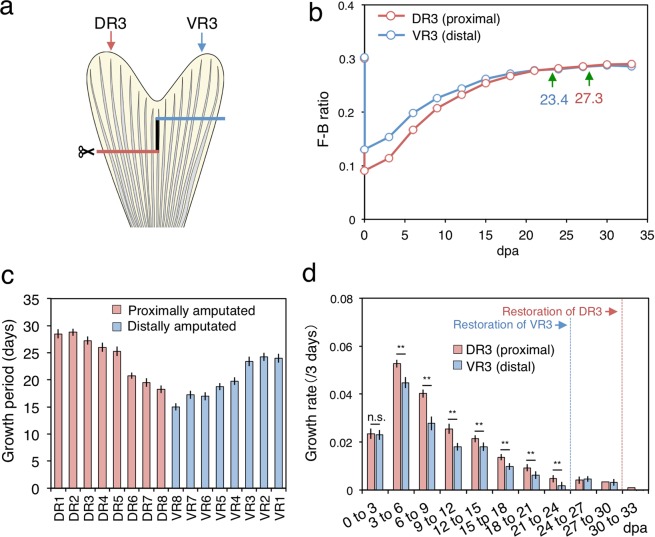
Growth analysis after stepwise (dorsal with proximal, and ventral with distal) amputation. (**a**) Schematic drawing of stepwise amputation. Dorsal fin rays (DR1-8) were amputated proximally and ventral fin rays (VR1-8) were amputated distally. (**b**) Transition of the F-B ratio in DR3 and VR3. Green arrows indicate the termination point for each fin ray. (**c**) Growth period of all fin rays. Red and blue bars indicate proximally and distally amputated fin rays, respectively. (**d**) Transition of the growth rate from 0 to 33 dpa in DR3 and VR3. Welch’s t-test: p-value ** < 0.01. n.s.: not significant. Error bars means ± SEM.

To test this hypothesis, taking advantage of the finding that fin rays symmetrically positioned in bi-lobes had similar growth periods and rates (Fig. 2c and Supplementary Fig. S8), we amputated fin rays of the dorsal and ventral lobes at different levels in the same specimen (stepwise amputation, n = 12, Fig. 4a). We amputated the dorsal fin rays (DR1-8) at a proximal level (the same level as in the straight amputation in Fig. 2a) and ventral fin rays (VR1-8) at a distal level (3 or 4 segments of lepidotrichia distal to the proximal amputation), and we tracked the F-B ratio to determine the growth period (Fig. 4b,c, Supplementary Fig. S10). For example, proximally amputated DR3 took 27.3 days to restore the fin ray length, but distally amputated VR3 terminated at 23.4 dpa (Fig. 4b). All of the fin rays had a shorter growth period after distal amputation than after proximal amputation (Fig. 4c, compare blue bars for the ventral lobe and red bars for the dorsal lobe), indicating that different levels of amputation did not result in the same growth period. In addition, reversed stepwise amputation (distal amputation in the dorsal lobe and proximal amputation in the ventral lobe, n = 9) showed similar growth periods as their distance counterparts in the original stepwise amputation (Supplementary Figs. S12a–c and S13). Thus, contrary to initial expectations, it appeared that the growth period was not necessarily fixed in each fin ray identity. In terms of growth rate, proximally amputated fin rays showed a faster growth rate than distally amputated ones (Fig. 4d, Supplementary Figs. S11 and S14). For example, in this experiment, proximally amputated DR3 had a faster growth rate than distally amputated VR3 throughout the growth period, with the same peak at 3 to 6 dpa (Fig. 4d). These results suggested that the more proximally a fin ray is amputated, the more the growth rate increases, in agreement with a previous study^17^, but the times needed to restore fin rays amputated at different depths are not the same, in contrast to the same previous study.

**Figure 5:**
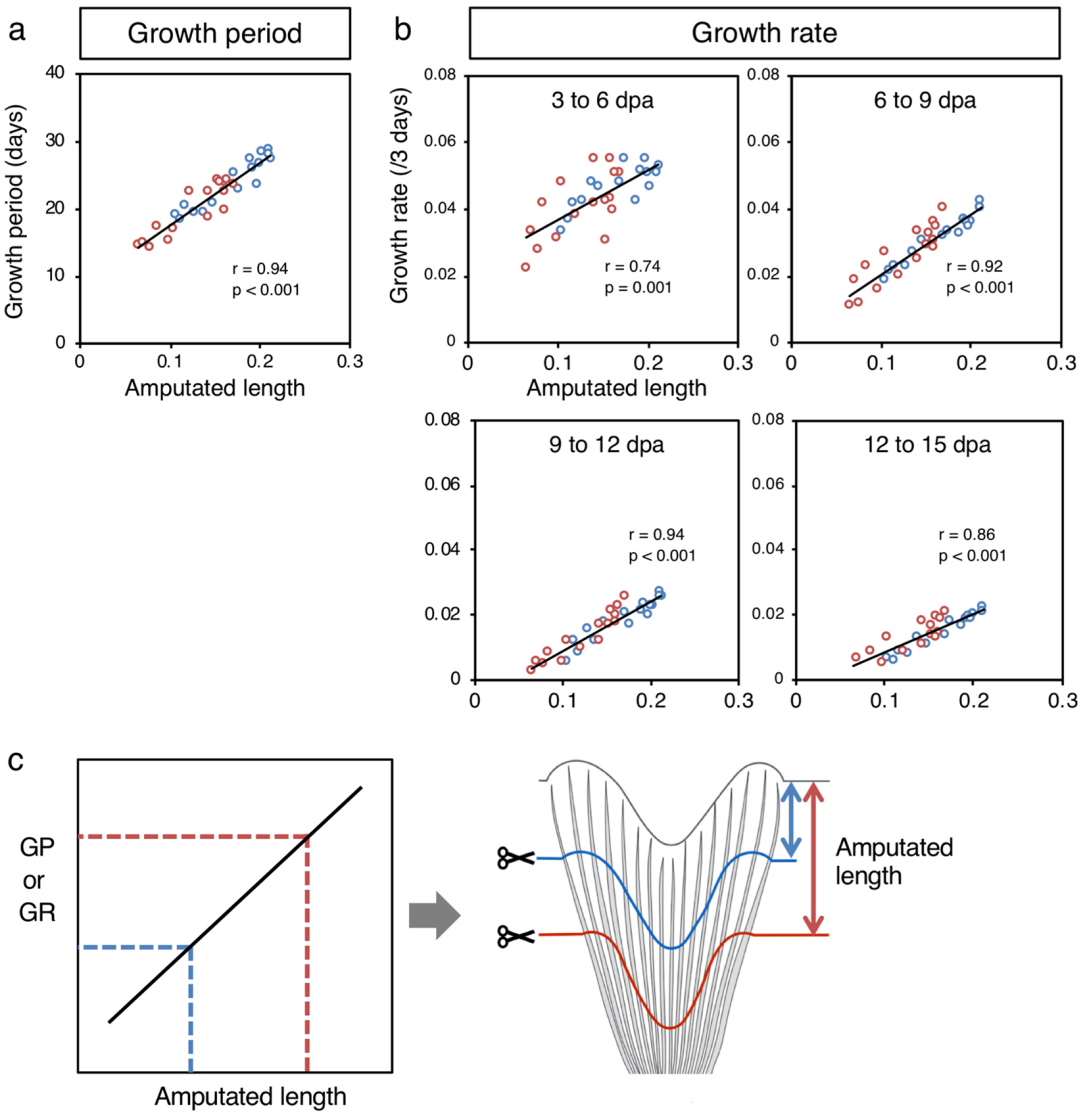
Correlation analysis between amputated length and growth period/rate. Correlation between the amputated length of the fin rays and the (**a**) growth period and (**b**) growth rate at 3 to 6 dpa, 6 to 9 dpa, 9 to 12 dpa, and 12 to 15 dpa. Red and blue circles indicate proximally and distally amputated fin rays, respectively. Black lines indicate linear regression. (**c**) The relationship of the amputated length of fin rays with the growth period and growth rate. Fin rays that were amputated the same distance from the tip of fin rays (right; red and blue lines) had the same growth rate and same growth period (left; dotted lines). The growth rate and growth period appeared to be determined by the amputated length regardless of the fin ray’s location. dpa: days post amputation. GP: growth period, GR: growth rate.

From our results above, we hypothesized that the growth period and rate are determined by the length (depth) of the amputation, regardless of the fin ray identity along the dorsal-ventral axis. To test this hypothesis, we conducted a correlation analysis between the amputated length of the fin ray and the growth period/rate in two types of stepwise amputation (with data in Fig. 4 and Supplementary Fig. S12). This analysis revealed that the growth period was significantly correlated with the amputated length of the fin ray (Fig. 5a), indicating that different levels of amputation do not result in the same growth period. We also analyzed the correlation between the amputated length of fin rays and the growth rate from 3 dpa to 15 dpa (the minimal termination points of the distally amputated DR8 in stepwise amputation), and we found that the growth rate was also significantly correlated with the amputated length of the fin ray (Fig. 5b). These results indicated that both the growth period and the rate of fin ray growth are determined by the amputated length of the fin ray, suggesting that fin rays amputated at the same distance from the tip of the fin ray show the same regrowth, regardless of their identity along the dorsal-ventral axis (Fig. 5c).

### Niche in the stump contributes to regrowth of regenerating fin ray

The results described above suggest that the same length of amputation had the same growth period/rate in the regenerating caudal fin rays. This means that, if the regenerating mesenchymal cells in the zebrafish caudal fin have a certain positional memory, the memory would not be the same between fin rays at the same proximal-distal level (Fig. 1b), but would be the same along a wavy line (Fig. 1c). This is inconsistent with the concept of positional memory that is supposed in the regenerating limb blastema, in which it is assumed that cells in the blastema at a certain proximal-distal level in the limb have a positional value (Fig. 1a).

We speculated that growth period and growth rate depend on conditions in the rest of the fin ray tissues near the amputation site (stump of a fin ray). To investigate what conditions (niche) in the stump (e.g., physical/physiological conditions, epigenetic situation of gene regulation) contribute to the control of regrowth of a regenerating fin ray, we focused on one physical condition, width (thickness) of fin rays, because a recent study^30^ showed that the thickness of fin rays correlates with the distance from the tip of the fin ray regardless of fin ray identity along the dorsal-ventral axis (DR1-8, VR1-8), and suggested that the girth of fin rays at the injury site as a niche determines the regenerated fin’s size and shape. Interestingly, that study showed that there is a wavy line of the same girth of fin rays in the unamputated caudal fin.

**Figure 6:**
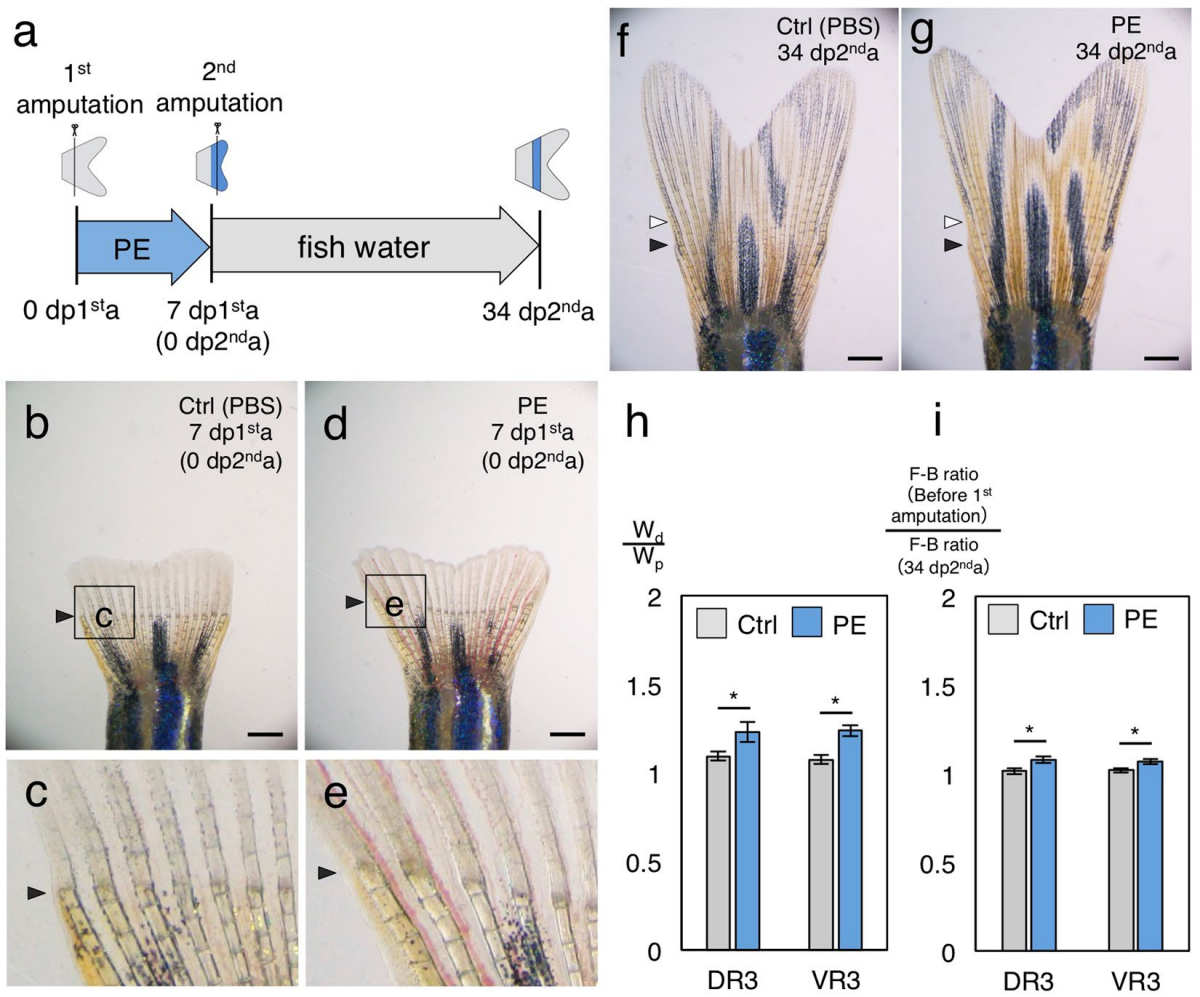
Scheme of phenylephrine treatment with twice fin amputations. (**a**) Scheme of the PE treatment. (**b**,**c**) Whole and magnified image of the control sample caudal fin on 7 dp1^st^a. (**d**,**e**) Whole and magnified image of PE-treated caudal fin on 7 dp1^st^a. (**f**) Whole image of the control sample fin on 34 dp2^nd^a. (**g**) Whole image of PE-treated fin of 34 dp2^nd^a. (**h**) Comparison of the width of fin ray at two segments of lepidotrichia distal to the first amputation site (W_p_) and proximal to the first amputation site (W_d_). (**i**) Comparison of F-B ratio on 0 dp1^st^a and 34 dp2^nd^a. Black and white arrowheads indicate first and second amputation sites, respectively. dp1^st^a: days post first amputation. dp2^nd^a: days post second amputation. PE: phenylephrine. Welch’s t-test: *p-value < 0.05. Error bars indicate standard error of measurement. Scale bars indicate 0.5 mm.

Phenylephrine (PE), a selective α1-adrenergic receptor agonist, is known to regulate proliferation of mouse osteoblast-like cells^31^. We found that PE treatment in regenerating fins sometimes gave rise to widened fin rays (DR3; n = 5/10, VR3; n = 4/10: Fig. 6b–e. See quantitative data in Fig. 6h). In addition, PE treatment during fin regeneration resulted in regeneration of the original length (Supplementary Fig. S15), suggesting that PE itself did not affect positional memory. To investigate whether the width of fin rays at the stump affects the length of the regenerated fin ray, we subsequently amputated the widened fin rays in the middle of the regenerated region at 7 days post first amputation (7 dp1^st^a; Fig. 6a). During the second regeneration, the amputated fins were not treated with PE. We found that the widened stump regenerated significantly longer fin rays than those before the first amputation (Fig. 6f,g,i). These results indicate the possibility that fin ray width at the amputation site contributes to the length of the regenerated fin ray.

## Discussion

### Reconstruction of the bi-lobed fin morphology in zebrafish caudal fin regeneration

To reconstruct the bi-lobed caudal fin morphology during regeneration, each fin ray must regrow to a certain length to restore its original length, depending on the fin ray identity along the dorsal-ventral axis (DR1-8, VR1-8). In straight-amputation experiments, we found that the longer fin rays in the lobe region had longer growth period and faster growth rate than the shorter fin rays in the cleft region. In addition, fin rays symmetrically positioned in the dorsal and ventral lobes that had a similar length had similar growth periods and rates. Furthermore, in stepwise amputation experiments, we found that proximally amputated fin rays had a longer growth period and faster growth rate than distally amputated fin rays, and these amputation-level-specific growth periods and rates were seen in all fin rays. Statistical analyses showed that both the growth period and growth rate were significantly correlated with the amputated length of the fin rays, suggesting that regardless of the fin ray identity along the dorsal-ventral axis, the growth period and rate of a fin ray are determined by the amputated length (Fig. 5c). In other words, amputated fin rays may recognize the length that has been removed and regulate both the growth period and rate to regenerate fin rays of the appropriate length, resulting in the reconstruction of the bi-lobed fin morphology.

### Termination of caudal fin regeneration

By tracking the F-B ratio, we could precisely evaluate the termination point of the restoration of the original fin ray length and describe the fin morphology reconstruction process. Our measurements revealed that the maximum period for restoring the fin ray length was 25.1 days for the longest fin ray, DR1 (Supplementary Fig. S6), after straight amputation. This growth period was approximately 1 week longer than previously thought^9^. In addition, the fin rays did not all terminate their restoration at the same time point, and the minimum growth period was 15.6 days in VR8, the shortest fin ray. The shortest ray regenerated around 10 days earlier than the longest ray. We succeeded for the first time in capturing the whole picture of the regrowth and restoration of fin length during caudal fin regeneration.

The time at which a restoring fin ray terminated its rapid growth is important for reconstructing the proper fin morphology. Artificial alteration of the rapid growth causes the overgrowth of restoring fin rays and the construction of a longer fin morphology that is different from the pre-amputation morphology^27^. In that study, the expression of *fa93e10*, a marker gene of fin growth, was correlated with the rapid fin growth^27^, and our expression analysis also showed a correlation between peak of growth rate and intensity of *fa93e10* expression. However, the *fa93e10* expression decreased to its pre-amputation level about 1 week before the estimated termination time point of the restored fin ray length. Thus, the *fa93e10* expression reflected the peak of growth rate during regeneration, but it did not appear to be appropriate as a criterion for the termination of morphological reconstruction. Similarly, our criterion based on the F-B ratio defines the end of the reconstruction of fin morphology but it does not necessarily mean completion of the whole regeneration process, such as anatomical restoration (segmentation and bifurcation of fin rays) and pigment pattern regeneration. There may be another indicator of termination, and to understand the whole process of organ regeneration, it is important to carefully determine the point of termination from various biological views.

### Positional memory in the regulation of fin ray length

To regenerate fin rays with the proper length, fin rays need to have some sort of recognition of their original length. This phenomenon can be explained by the concept of “positional memory”, which has been well-studied in amphibian limb regeneration. In limb regeneration, limb blastema cells are thought to have positional memory along the proximal-distal axis that provides information about their position, i.e., information about where the cells are relatively located in the limb (Fig. 1a, number 3 or 5 at the site of amputation) and what the cells need to restore (Fig. 1a, numbers 4-7 or 6–7 in the regenerated limb). Many studies have shown evidence of positional memory^2–8^. For example, when the blastema formed in a distally amputated limb is transplanted to a more proximally amputated limb, the grafted blastema only contributes to the formation of the structures distal to the originally amputated site^3,4^. This suggests that positional memory reactivated in the blastema is never affected by its surroundings. Importantly, cells at an amputation point along the proximal-distal axis have the same positional memory in a blastema at the proximal-distal point (number 3 and 5 in Fig. 1a).

Applying the concept of positional memory in amphibian limb regeneration to caudal fin regeneration in zebrafish, a proximal amputation (number 3 in Fig. 1b) gives rise to a longer regeneration (numbers 4–7), while a distal amputation (number 5 in Fig. 1b) results in a shorter regeneration (numbers 6–7). Some studies in zebrafish have suggested that fin tissues also possess positional memory. Grafting a fin ray or a hemi-ray to a different fin region and subsequent amputation of the grafted fin ray results in a restored length similar to the original length of the donor fin region rather than that of the host fin region^14,15,24^. These results suggest that the length of a restored fin ray is determined by some kind of positional memory along the proximal-distal axis. Shibata *et al*.^24^ found that the blastema cells that form at the tip of distally amputated fin rays and are transplanted to the tip of proximally amputated fin rays contribute to forming an entire regenerated fin ray, regardless of the proximal-distal level of the blastema cells. This does not correspond to the above-described characteristics of blastemal positional memory in amphibians. Based on these results, the authors^24^ suggested that not the blastema cells, but rather the stump tissue at the amputated site, possesses positional memory.

In this study, we found that the growth period and rate in fin regeneration are significantly correlated with the amputated length (depth) of the fin ray (Fig. 5a,b), suggesting that the fin rays use information not about fin ray identity along the dorsal-ventral axis (DR1-8, VR1-8; information regarding the original length of each fin ray) but about the amputated length for restoration of the original length. This idea is similar to the concept of positional memory along the proximal-distal axis of the caudal fin. However, our findings also indicated that fin rays amputated with the same lengths (blue and red lines in Fig. 5c) have the same growth periods and rates regardless of the fin ray identity. If each fin ray used its own memory to recognize the amputated length, the same memory would be not on a straight line (Fig. 1b) but rather on a wavy line along the dorsal-ventral axis (Fig. 1c). This does not correspond to the presumable positional memory in the blastema that is supposed to be the same at the proximal-distal level of the fin (Fig. 1b), as in amphibian limb regeneration (Fig. 1a).

In addition, there is the possibility that structural/physical conditions (niche) at the amputated site of the stump tissue could be used as memory to recognize the amputated length of the fin ray, as suggested by a recent study^24^. Interestingly, a recent study showed that there is a tight correlation between fin ray length and fin ray thickness (girth at a position of a fin ray)^30^, which corresponds to our results for growth period/rate. It is possible that the thickness of the fin ray may contribute to the determination of regenerated fin ray length as a memory of the stump niche. In the present study, amputation of widened fin rays made by PE treatment gave rise to regeneration of longer fin rays (Fig. 6f–h). These results support the idea that positional memory in not the blastema but in the niche at the stump, such as the width of fin rays, may determine the length of the regenerated fin ray. Further studies should be carried out to reveal the mechanism by which the length that an amputated fin ray regenerates is determined.

## Methods

### Zebrafish husbandry

All zebrafish experiments were conducted in accordance with relevant nation and international guidelines, and were approved by the Tohoku University Animal Research Committee (Permit Number: 2018LsA-015). RIKEN wild-type strain adult zebrafish (six-month-old in the growth period analysis, three-month-old in the qRT-PCR and Whole-mount *in situ* hybridization analysis, and four-month-old in phenylephrine treatment) were used in all experiments. Individuals in similar age had different body size, but growth period in fin regeneration seemed similar (see Supplementary Fig. S7). Fish were maintained at 27 °C, 14-hours bright/10-hours dark.

### Body and fin ray length measurements and F-B ratio calculation

Body and fin ray length were measured as described below. The whole body and caudal fin were photographed with a camera (Pixera Penguin 600CL and Olympus DP74) connected to a stereoscopic microscope (Leica M165 FC). To reduce variance in the measured length from the photographs, the photograph capture and arrangement were repeated five times. ImageJ was used to measure the length of the body and fin from the photographs and the mean of five measurements was used in the analysis. Body length was measured from the upper jaw to the distal tip of the caudal peduncle^26^ (see Supplementary Fig. S2a). Fin ray length was measured from the caudal peduncle to the distal tip of the caudal fin parallel to each fin ray^26^ (see Supplementary Fig. S2b,c). F-B ratio on day N was calculated as fin length (day N)/Body length (day N).

### Fin amputation

Fish were anesthetized by 0.02% tricaine (Tokyo Chemical Industry) in 0.03% artificial seawater. Caudal fins were amputated with a razor. The amputation site was as follows. Straight amputation: parallel to the dorsal-ventral axis at the tip of a non-segmented fin ray that was connected to the uroneural. Stepwise amputation: the dorsal and ventral fin rays were amputated proximally or distally, respectively. proximal amputation was at the same site as the straight amputation, and distal amputation was at three to four segments of lepidotrichia distal to the proximal amputation.

### qRT-PCR

cDNA was synthesized from total RNA, which was extracted in three regions (Dorsal lobe: DR3 to DR5, Cleft region: DR7 and DR8, VR7 and VR8, Ventral lobe: VR3 to VR5) in four fins including blastema and two segments of fin rays. RNA was extracted by RNeasy plus micro kit (QIAGEN). cDNA was synthesized by reverse transcription with SuperScript III (Invitrogen). qRT-PCR was performed by StepOnePlus (Applied Biosystems) with Power SYBR Green PCR Master Mix (Applied Biosystems). Quantitative analysis of *fa93e10* and *ef1a* was repeated three times for each time point. *fa93e10* experiments were normalized by *ef1a* amount. The following primers were used for qRT-PCR: *fa93e10* forward primer AGT GGC AGT AAT GAG CAG GC and reverse primer GCT GGG TAT CAA CAG GAG CA; *ef1a* forward primer TCC TCT TGG TCG CTT TGC TG and reverse primer GTG TGA TTG AGG GAA ATT CAC TTG.

### Whole-mount *in situ* hybridization (*fa93e10*)

The primer sequences used to make RNA probes of *fa93e10* were described in Kujawski *et al*. Fins were fixed in 4% paraformaldehyde (PFA) in phosphate-buffered saline (PBS) overnight at 4 °C and washed twice in PBS for 10 minutes at 4 °C. The fins were then dehydrated in a methanol/PBS + 0.01% Tween20 (PBT) series (25% methanol/PBT, 50% methanol/PBT, 75% methanol/PBT) and 100% methanol for 10 minutes each. The fins were stored in 100% methanol at −20 °C until the next treatment. Next, the fins were rehydrated in a methanol/PBT series (75% methanol/PBT, 50% methanol/PBT, 25% methanol/PBT) at 4 °C for 10 minutes each. After rehydration, the fins were re-fixed in 4% PFA/PBS for 30 minutes at 4 °C and washed in PBT twice for 10 minutes each at 4 °C. The fins were then incubated in 2.25 μg/ml Proteinase K (Invitrogen)/PBT for 10 minutes and washed twice in PBT for 10 minutes each at 4 °C. After two washes with PBT, the fins were re-fixed in 4% PFA/PBS for 30 min at 4 °C and washed in PBT twice for 10 min at 4 °C. The fins were then incubated twice in pre-hybridization mix (50% formamide, 5 × SSC, 0.1% Tween20, 50 µg/ml *E*. *coli* tRNA, 50 µg/ml heparin) for 5 minutes and over an hour, respectively, at 65 °C. After the two incubations with pre-hybridization mix, the fins were incubated in hybridization mix (1 µg/ml digoxigenin-labeled RNA probe diluted in pre-hybridization mix) overnight at 65 °C. The fins were then washed twice in 50% formamide/2 × SSCT (SSC + 0.1% Tween 20) for 30 minutes at 65 °C, 2 × SSCT for 15 minutes at 65 °C, and twice in 0.2 × SSCT for 30 minutes at 65 °C. The fins were then incubated in 10% inactivated goat serum twice, for 10 minutes and over an hour, at room temperature (RT). The fins were then incubated with 0.025% Anti-Digoxigenin-AP Fab fragments (Roche) diluted in 10% inactivated goat serum overnight at RT, and washed four times in 10% inactivated goat serum for 25 minutes at RT. The fins were then incubated four times in staining buffer (100 mM Tris HCl (pH 9.5), 50 mM MgCl_2_, 100 mM NaCl, 0.1% Tween 20, 1 mM levamisol) for 5 minutes at RT. After four incubations with staining buffer, the fins were incubated with substrate in staining buffer (substrate: 4.5 µl/ml NBT, 3.5 µl/ml BCIP) for over an hour at RT and washed twice in PBS for 10 minutes each. Samples were observed and photographed in the same solution with a camera (Pixera Penguin 600CL) connected to a stereoscopic microscope (Leica M165 FC).

### Phenylephrine treatment

A 1 M solution of Phenylephrine Hydrochloride (Wako Pure Chemical Industries) dissolved in PBS was stored in the dark at 4 °C, and diluted to 1 mM for use in the experiments. Fins were first amputated following the same method as for straight amputation (see Fin amputation in Methods). In the second amputation, regenerated fin rays were amputated at two to three segments of lepidotrichia distal to the first amputation site. After the first caudal fin amputation, fish were housed in fish water (five fish/litter) with 1 mM PE solution or with the same amount of PBS. PE or PBS containing water was changed every day. After the second caudal fin amputation, fish were housed in fish water (five fish/litter) and the water was changed every 3 days.

### Statistical analysis

All statistical analysis was performed using EZR statistical software^32^. Statistical differences between two groups were analyzed by Welch’s t-test or paired t-test. Pearson’s correlation coefficient was used to measure correlations between two groups.

## Supplementary information

Supplementaryinformation.
