# Supplementary material for: Regrowth of zebrafish caudal fin regeneration is determined by the amputated length
Source: Sci Rep. 2020 Jan 20;10:649. doi: 10.1038/s41598-020-57533-6 (PMC6971026; doi:10.1038/s41598-020-57533-6)
Supplement: Supplementary file 1 — Supplementaryinformation. [file 41598_2020_57533_MOESM1_ESM.pdf]

Supplementary Information

Regrowth of zebrafish caudal fin regeneration is determined by the amputated length.

Toshiaki Uemoto[1], Gembu Abe[1]*, Koji Tamura[1]

[1] Laboratory of Organ Morphogenesis, Department of Ecological Developmental Adaptability Life Sciences, Graduate School of Life Sciences, Tohoku University, Aobayama Aoba-ku, Sendai 980-8578, JAPAN.

* Corresponding author.
E-mail: gembu.abe.b5@tohoku.ac.jp
Phone: +81-22-795-6677
Fax: +81-22-795-6677

Supplementary Methods

Numbering the fin rays

Terminology for the fin skeleton used in this study is in accordance with previous studies[10,11,13]. We wanted to use the minimum number of fin rays necessary that still took the heart-shape morphology of the fin into consideration. For this purpose, we selected 16 principle fin rays that have similar bone segment structure. We did not use procurrent rays to avoid deep amputation (Supplementary Fig. S1**a**).

We first defined the number and identity of the fin rays in the sample because the number of fin rays in the caudal fin can vary between individuals [16 to 18 fin rays per fish in the present study (n=19)]. To identify each fin ray along the dorsal-ventral axis, we observed the connection of the base of the fin ray to the endoskeleton of the caudal fin (hemal spine, par-hypural, and hypurals) and found that the number of fin rays connecting to each endoskeleton was different between specimens (Supplementary Fig. S1**a**). In the dorsal lobe, two or three fin rays were connected to hypural 5, the dorsal-most hypural. Two or three fin rays were connected to hypural 4, and three or four fin rays were connected to hypural 3. In the ventral lobe, two or three fin rays were connected to hypural 2, two or three fin rays were connected to hypural 1, and one or two fin rays were connected to the par-hypural. Only one fin ray was connected to the hemal spine.

Given the variance in the number of fin rays connected to each endoskeleton, we selected the fin rays that were present in more than 80% of the specimens (Supplementary Fig. S1**a**, **b**): two fin rays for hypural 5, three for hypural 4, and three for hypural 3, and we named them DR1-DR8 from dorsal to ventral. In the ventral lobe of more than 80% of the specimens, there were two fin rays for hypural 2, three for hypural 1, two for the par-hypural, and one for the hemal spine. We named these eight fin rays VR1-VR8 from ventral to dorsal. We used these 16 fin rays (DR1-DR8 and VR1-VR8) for length measurements. When there were remaining fin rays on the endoskeleton, we omitted the ventral ones for each hypural and always used only 16 fin rays.

Definition of termination point and growth parameters

To evaluate whether fin ray growth becomes constant, we investigated the F-B ratio of non-amputated fin rays. The F-B ratio of each fin ray was not completely constant and there were slightly different F-B ratios at different time points in non-

amputated caudal fins (Supplementary Fig. S3**b** for DR3, see also Supplementary Fig. S4). The F-B ratios slightly increased and decreased between time points; therefore, we determined a range of F-B ratios for the period of fin ray constant growth (green shading in Supplementary Fig. S3**b**). For this purpose, we calculated the changes in F-B ratios between two successive time points as the growth rate of the pre-amputation state. Growth rate was calculated as the F-B ratio on day N+3 minus the F-B ratio on day N. To determine the range of growth rates in non-amputated fins, we recorded the F-B ratio every 3 days for 1 month (from day 0 to day 33). We then detected and removed the outlier values as determined by the Smirnov-Grubbs Test. To evaluate the "constant state of growth" precisely, we collected two successive growth rates and plotted them (Supplementary Fig. S3**c** for DR3, see also Supplementary Fig. S5). In this graph, the growth rate between day N and day N+3 (GR1) is plotted on the vertical axis, and the next growth rate between day N+3 and day N+6 (GR2) is plotted on the horizontal axis. During all periods in 1 month, the combinations of GR1 and GR2 formed a 95% confidence ellipse (green ellipse in Supplementary Fig. S3**c** for DR3, see also Supplementary Fig. S5), and we evaluated this area as the range of the constant-state growth rate. Finally, we regarded the termination point of the restoration of fin ray length as the time point when a combination of two successive growth rates of a regenerating fin ray became included in the 95% confidence ellipse or were in the area for less than 0 (the whole green area in Supplementary Fig. S3**c** for DR3, see also Supplementary Fig. S5).

Supplementary Figures

a

	Hypural5	Hypural4	Hypural3	Hypural2	Hypural1	Par-hypural	Hemal spine
Fluctuation	2 ~ 3	2 ~ 3	3 ~ 4	2 ~ 3	2 ~ 3	1 ~ 2	1
> 80%	2	3	3	2	3	2	1

b

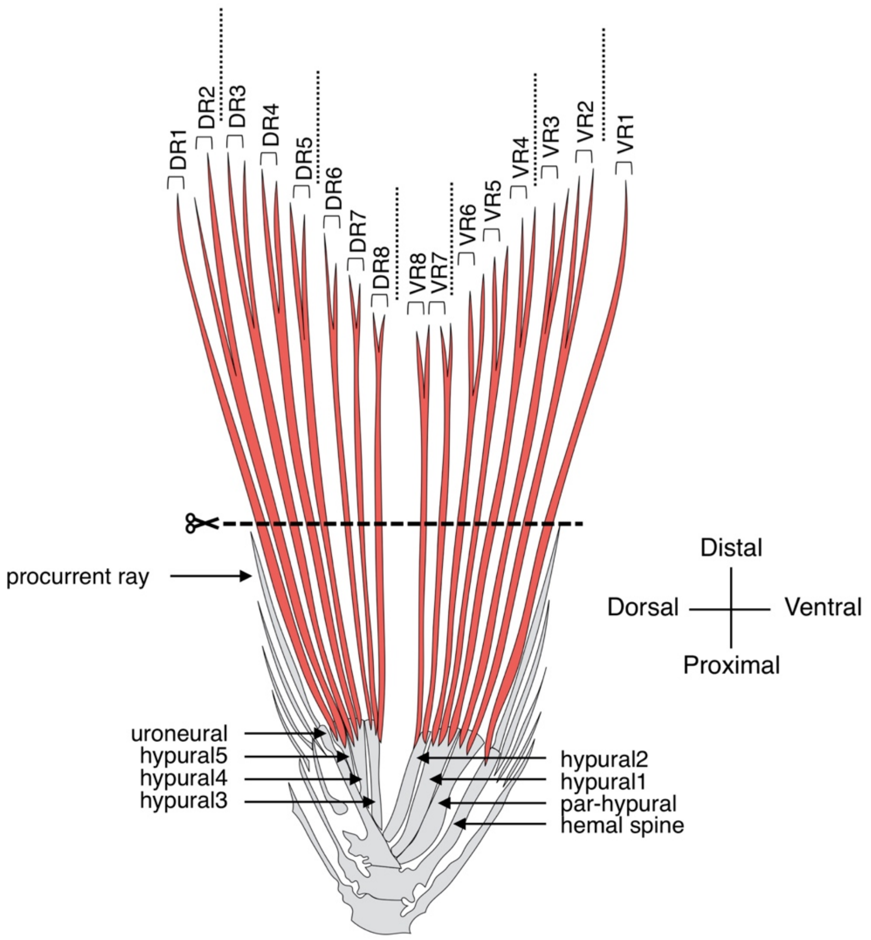

Supplementary figure 1. The number of fin ray and schematic representation of the caudal fin skeleton.

(a) Fluctuating number and the high (more than 80%) common number of fin rays connected to each endoskeleton. (b) Schematic of the caudal fin skeleton. Sixteen fin rays that are highly common in fish, are colored red: 8 dorsal fin rays (DR1-8) and 8 ventral fin rays (VR1-8). The amputation site for the straight amputation is indicated as a dotted line, at the tip of a pro-current fin ray.

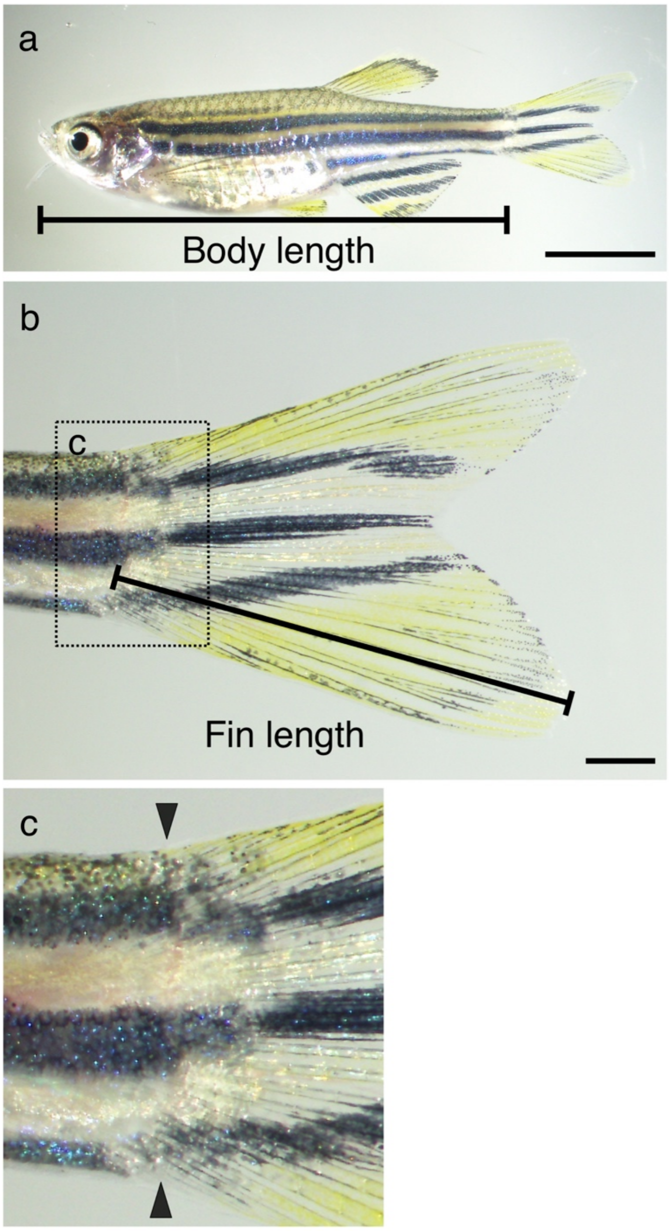

Supplementary Figure S2. Measurements of body length and fin ray length.
(a) A specimen for measuring body length and fin length. Body length represents length from the tip of the upper jaw to the caudal peduncle. (b) Fin ray length represents length from the caudal peduncle to the tip of the fin ray. (c) Higher magnification shows the position of the boundary between the body and caudal fin used in this study (arrowheads). The caudal peduncle is at the posterior end of body muscle. Scale bars in (a) and (b) indicate 5 mm and 1 mm, respectively.

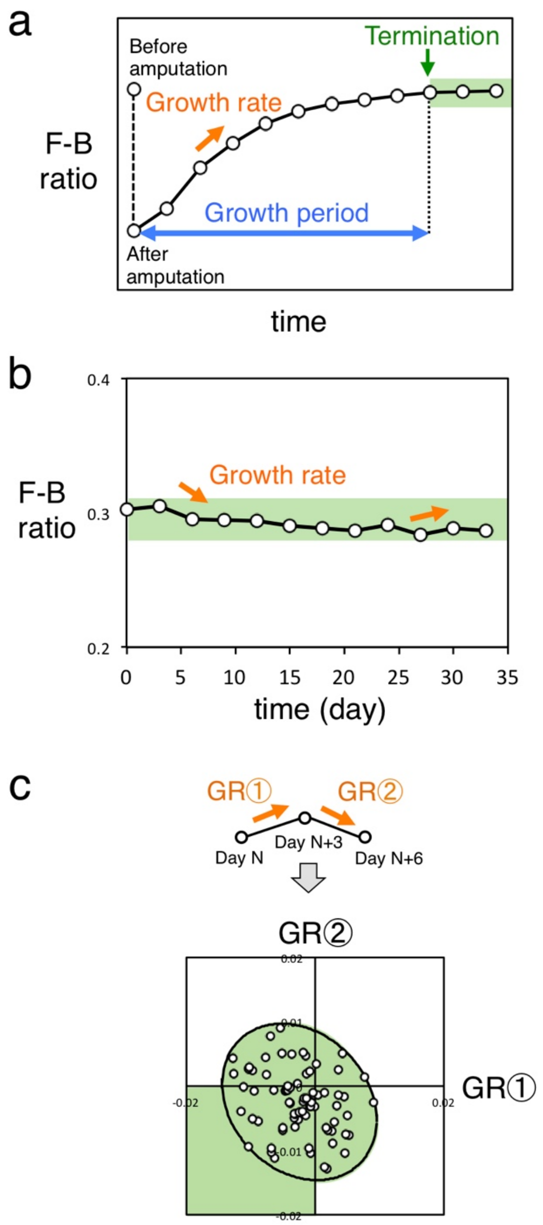

Supplementary figure 3. F-B ratio in amputated and non-amputated fin.

(**a**) Schematic drawing of the transition of the F-B ratio for an amputated fin. The F-B ratio increases in the fin-amputated fish and eventually become constant (green region). (**b**) The actual F-B ratio in a non-amputated fin ray (DR3). The F-B ratio is almost constant but fluctuates slightly. (**c**) Combinations of two successive growth rates of a fin ray (DR3). The growth rate on day N to day N+3 and the growth rate of day N+3 to day N+6 in a non-amputated DR3 were recorded, and the range of growth rates was analyzed as a 95% confidence ellipse. GR: growth rate. Error bars means ± SEM.

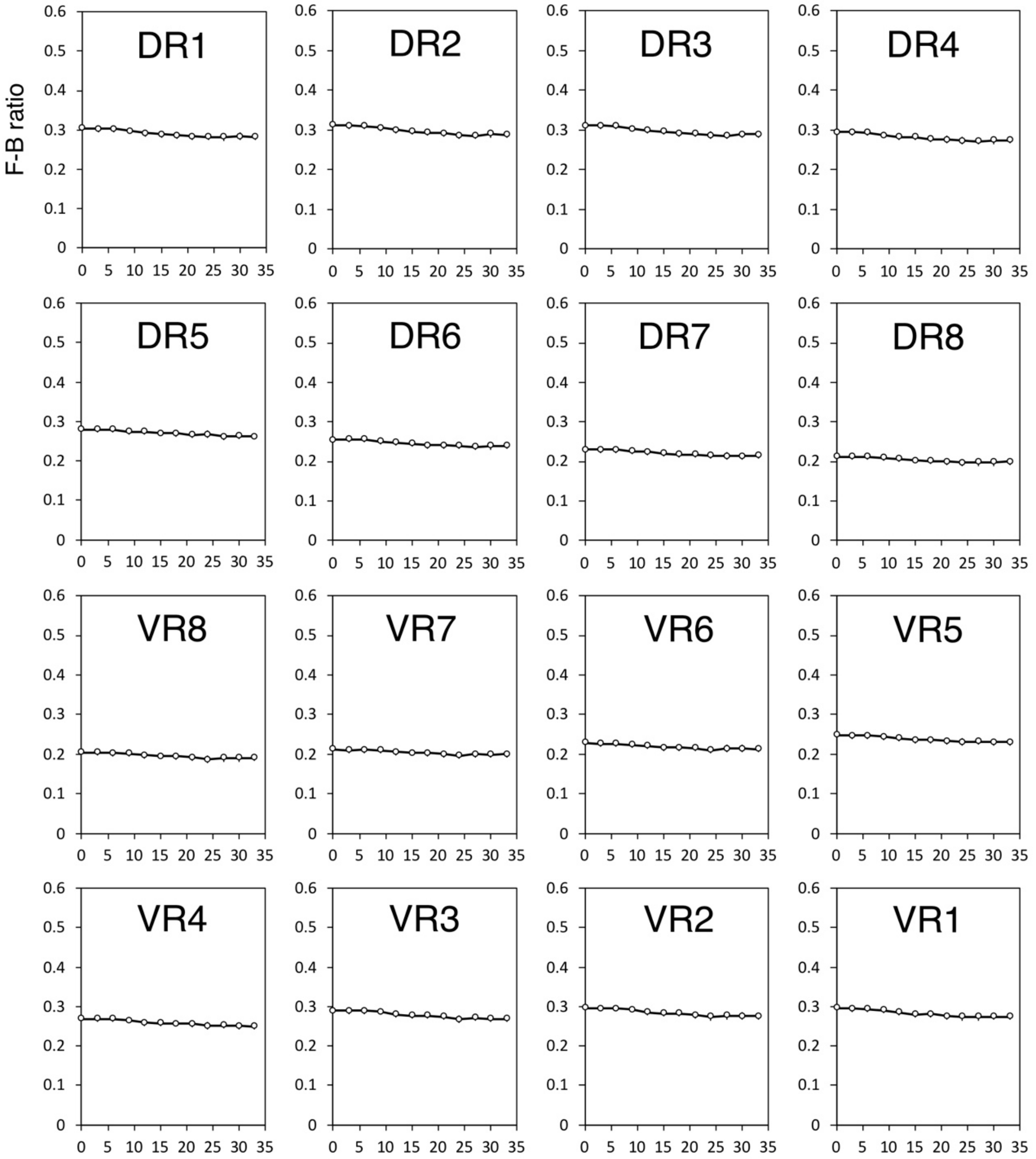

Supplementary figure 4. F-B ratio for all non-amputated fin rays.

The F-B ratio for each non-amputated fin ray (DR1-8, VR1-8) was traced from day 0 to day 33. Error bars means ± SEM.

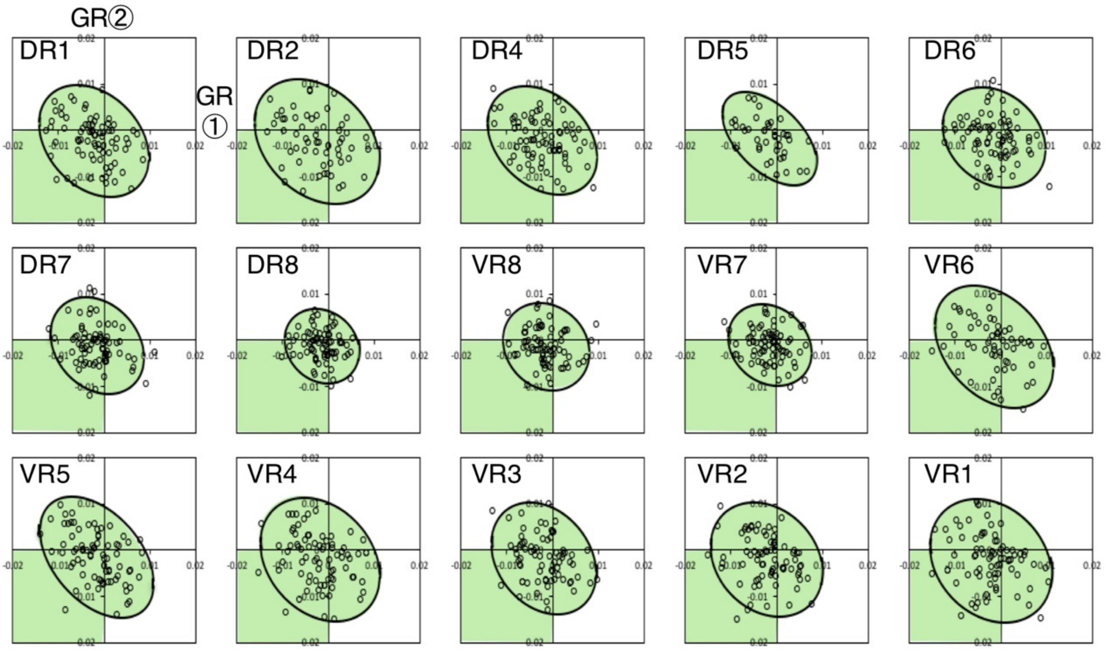

Supplementary figure 5. Combinations of two successive growth rates for non-amputated fin rays.

From the fluctuation of the F-B ratio for non-amputated fin, a confidence ellipse of two successive growth rate was constructed. The difference in the F-B ratio between day N and day N+3 and between day N+3 and day N+6, were calculated. The criterion for regeneration termination is colored green GR: growth rate.

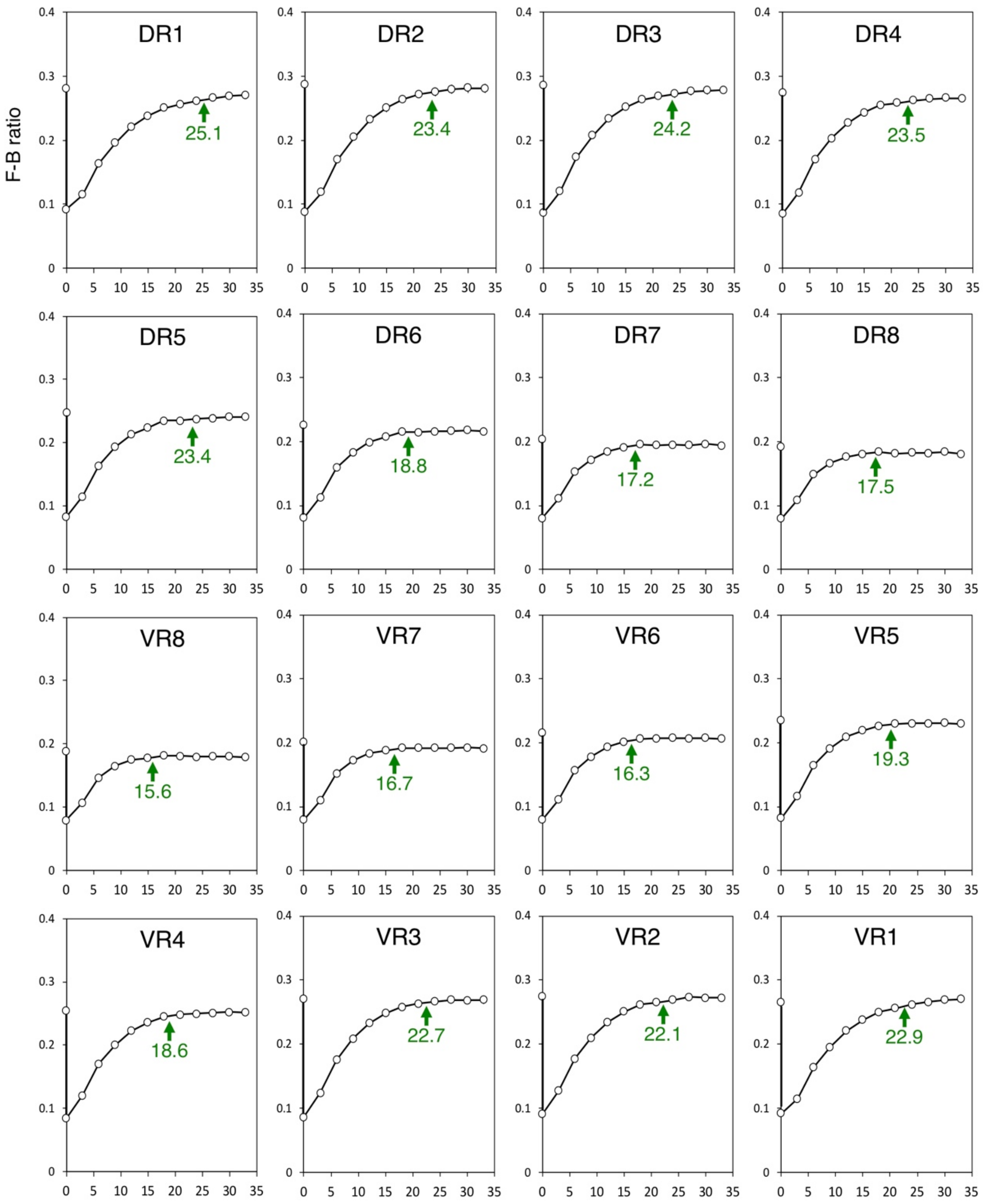

Supplementary figure 6. F-B ratio transition after straight-amputation

Transition of the F-B ratio of each fin ray. Green arrows indicate termination points. Error bars means ± SEM.

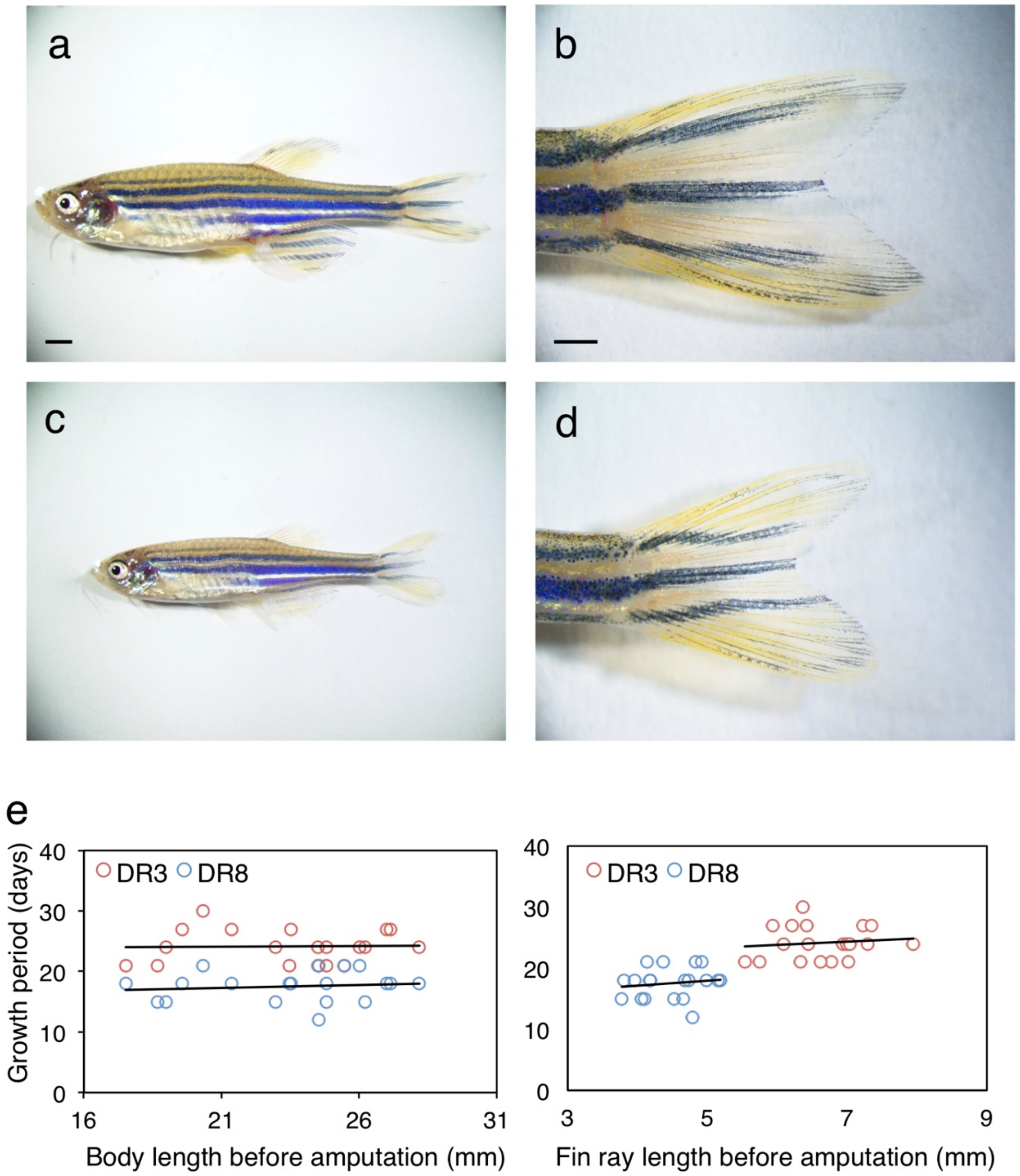

Supplementary Figure S7. Growth period is independent of body and fin size. (a)-(d) Whole body and caudal fin of a large and small fish. (a) Whole body of a large fish. (b) Whole fin of a large fish. (c) Whole body of a small fish. (d)Whole fin of a small fish. (e) Growth periods of specific fin rays (DR3, red circles; DR8, blue circles) in fish with various body and fin lengths. Black lines indicate linear regression. Scale bars in (a) and (b) indicate 2 mm and 1 mm, respectively.

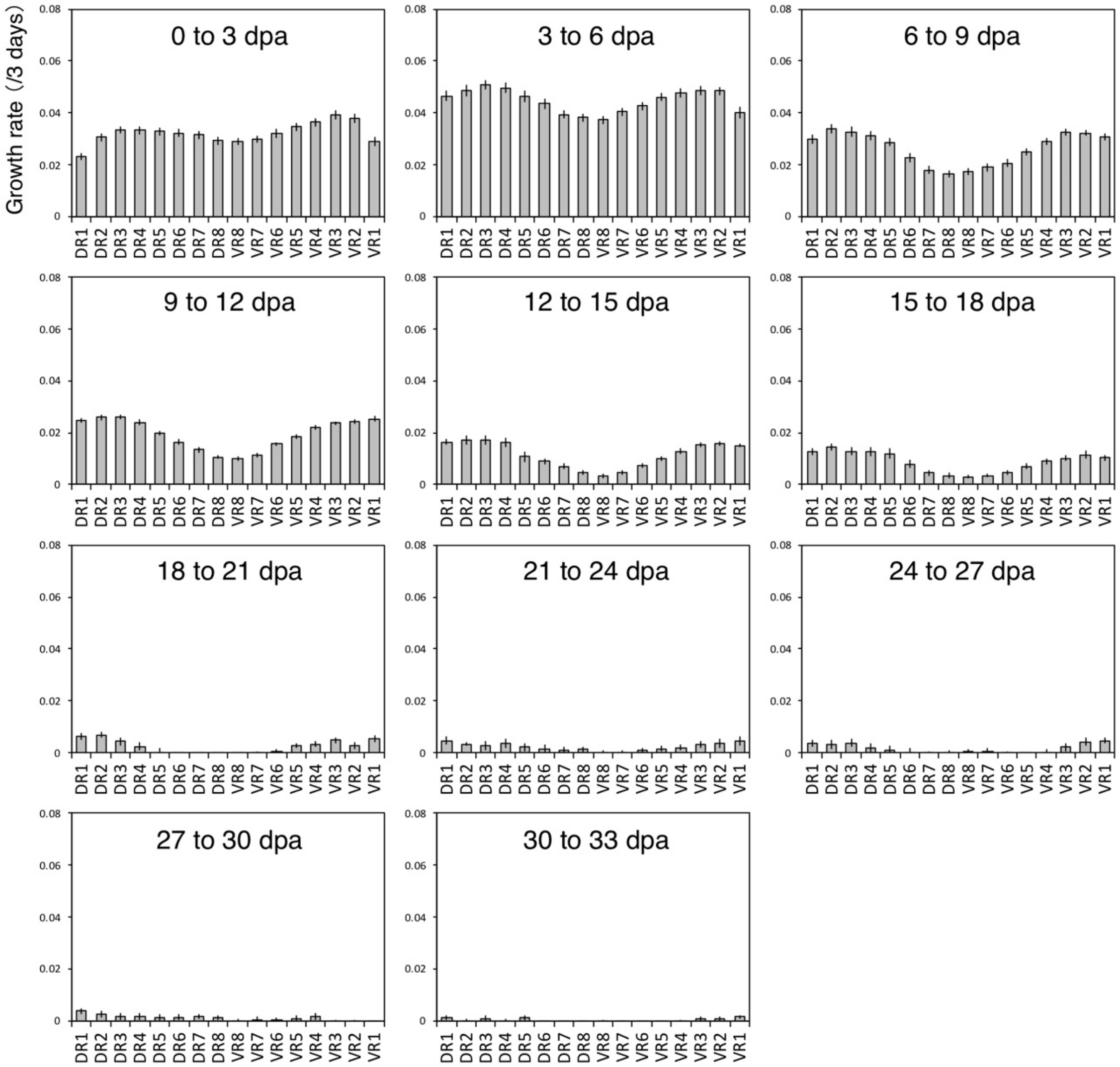

Supplementary figure 8. Growth rate of straight amputated fin rays during each growth period. The growth rate from 0 to 33 days post-amputation (dpa) was calculated every three days as the difference in the F-B ratio between N dpa and N+3 dpa. Error bars means ± SEM.

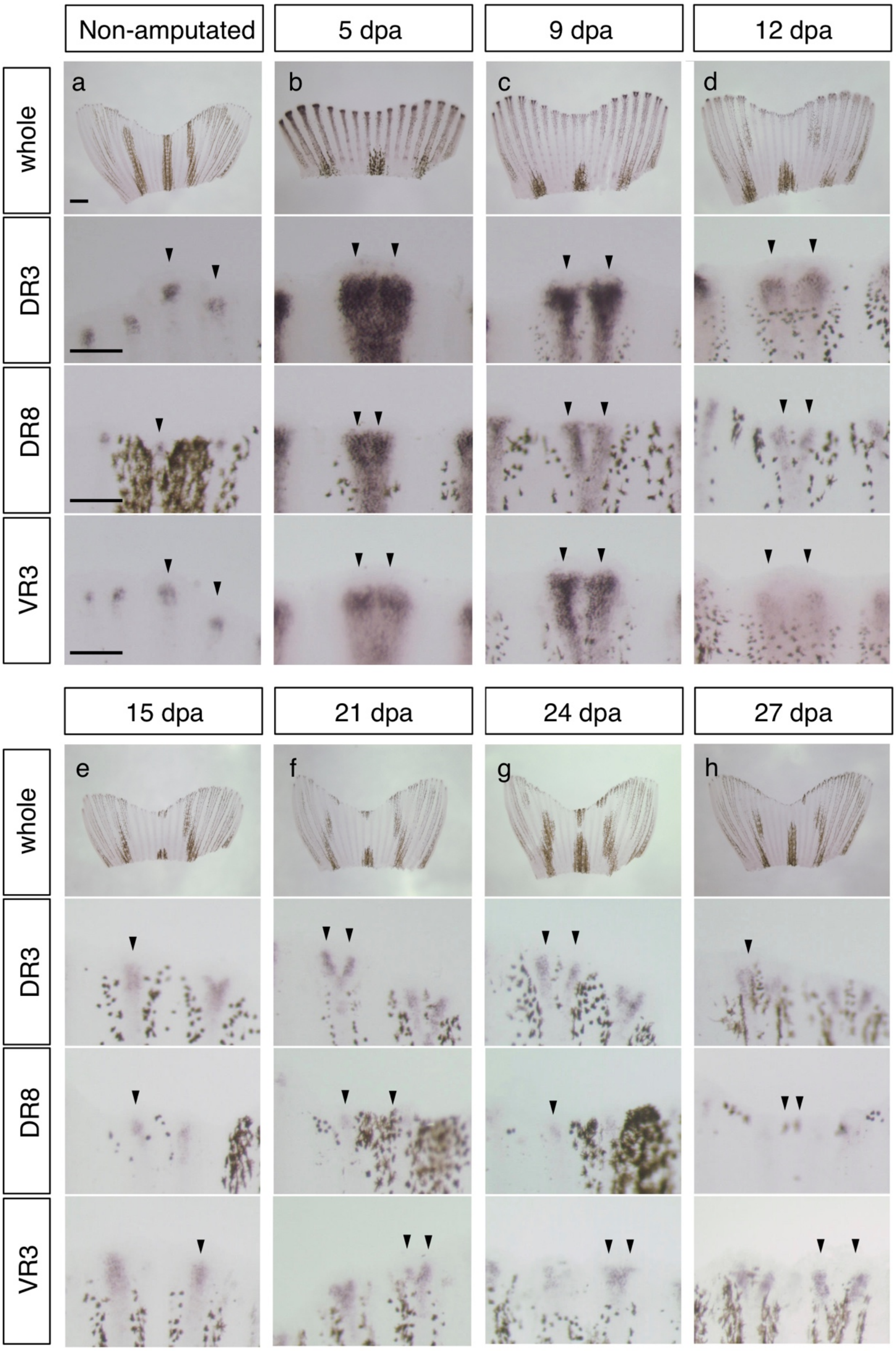

Non-amputated
5 dpa
9 dpa
12 dpa
whole
DR3
DR8
VR3
a
b
c
d
15 dpa
21 dpa
24 dpa
27 dpa
e
f
g
h

Supplementary figure 9. *In situ* hybridization of *fa93e10* in a non-amputated and amputated fin.

Whole fin image and magnified images of DR3, DR8, and VR3. In the non-amputation state, the *fa93e10* expression was low (n = 3/4) or undetectable (n = 1/4) at the distal tip of each fin ray. After amputation, all of the fish (5 dpa, n = 4; 9 dpa, n = 5; 12 dpa, n = 6; 15 dpa, n = 4; 21 dpa, n = 4; 24 dpa, n = 5; 27 dpa, n = 4) showed *fa93e10* expression in the tip of the caudal fin rays The expression became highest at 5 dpa in all of the fin rays and the high expression continued until 12 dpa. Arrowheads indicate the expression of *fa93e10*. When a fin ray was bifurcated, both of the fin rays are indicated by the arrowheads. Scale bars indicate 0.5 mm.

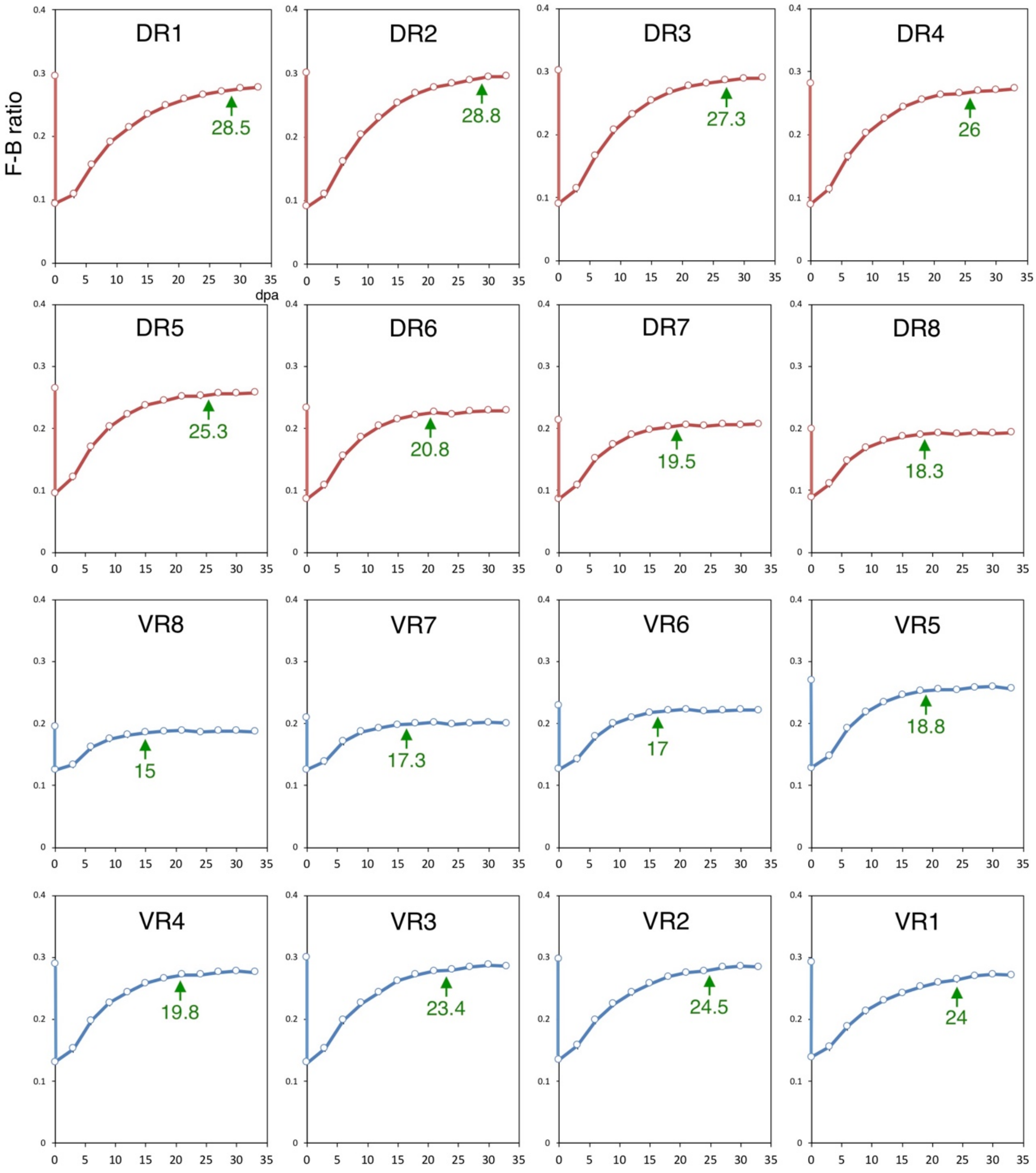

Supplementary figure 10. F-B ratio transition after stepwise (dorsal with proximal, and ventral with distal) amputation

Transition of the F-B ratio of each fin ray. Green arrows indicate termination points. After termination, the F-B-ratio was almost constant. Red and blue lines indicate proximally and distally amputated fin rays, respectively. Error bars means ± SEM.

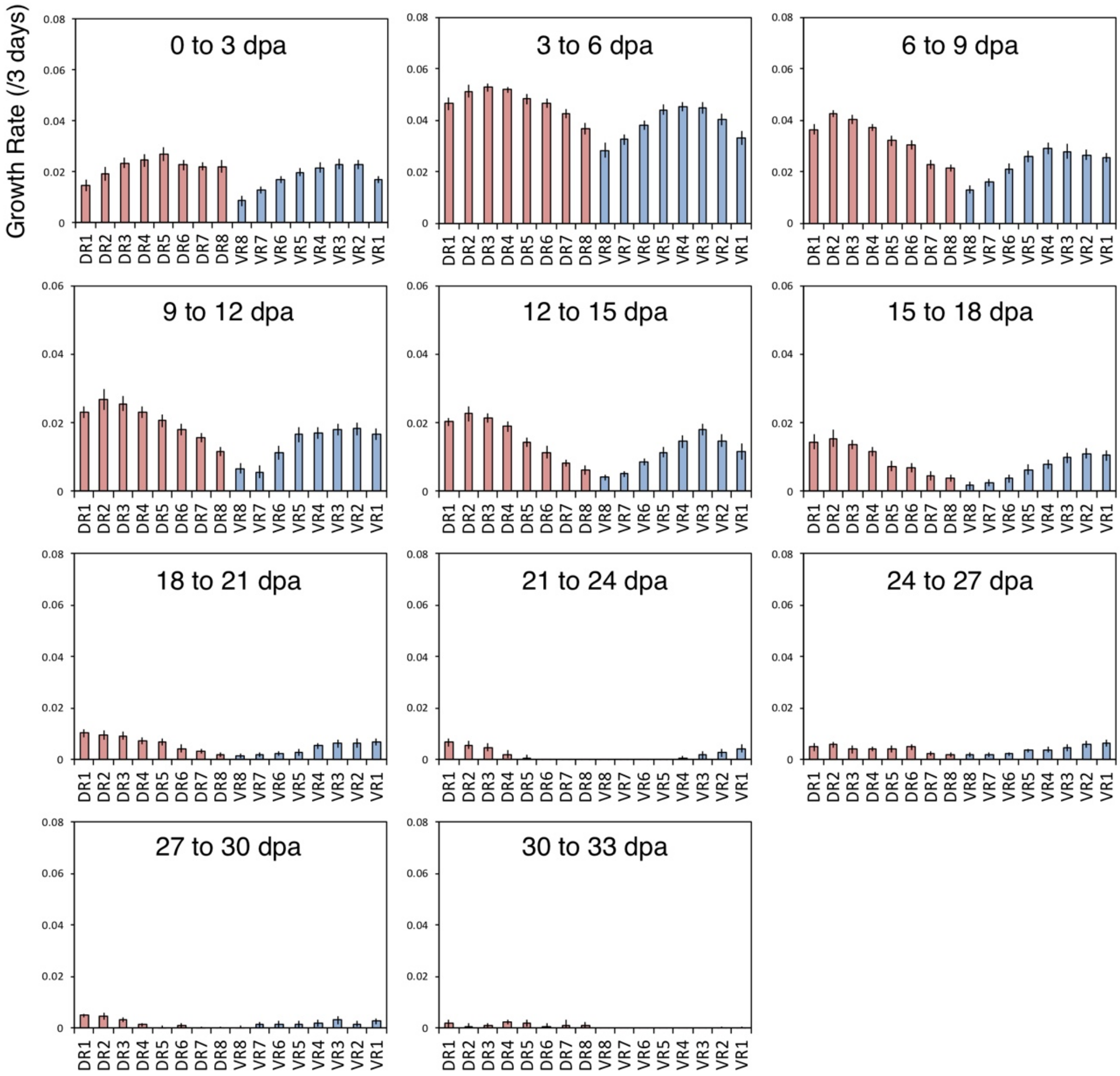

Supplementary figure 11. Growth rate of stepwise (dorsal with proximal, and ventral with distal) amputated fin rays during each growth period.

The growth rate from 0 to 33 dpa was calculated every three days. Red and blue bars indicate proximally and distally amputated fin rays, respectively. Error bars means ± SEM.

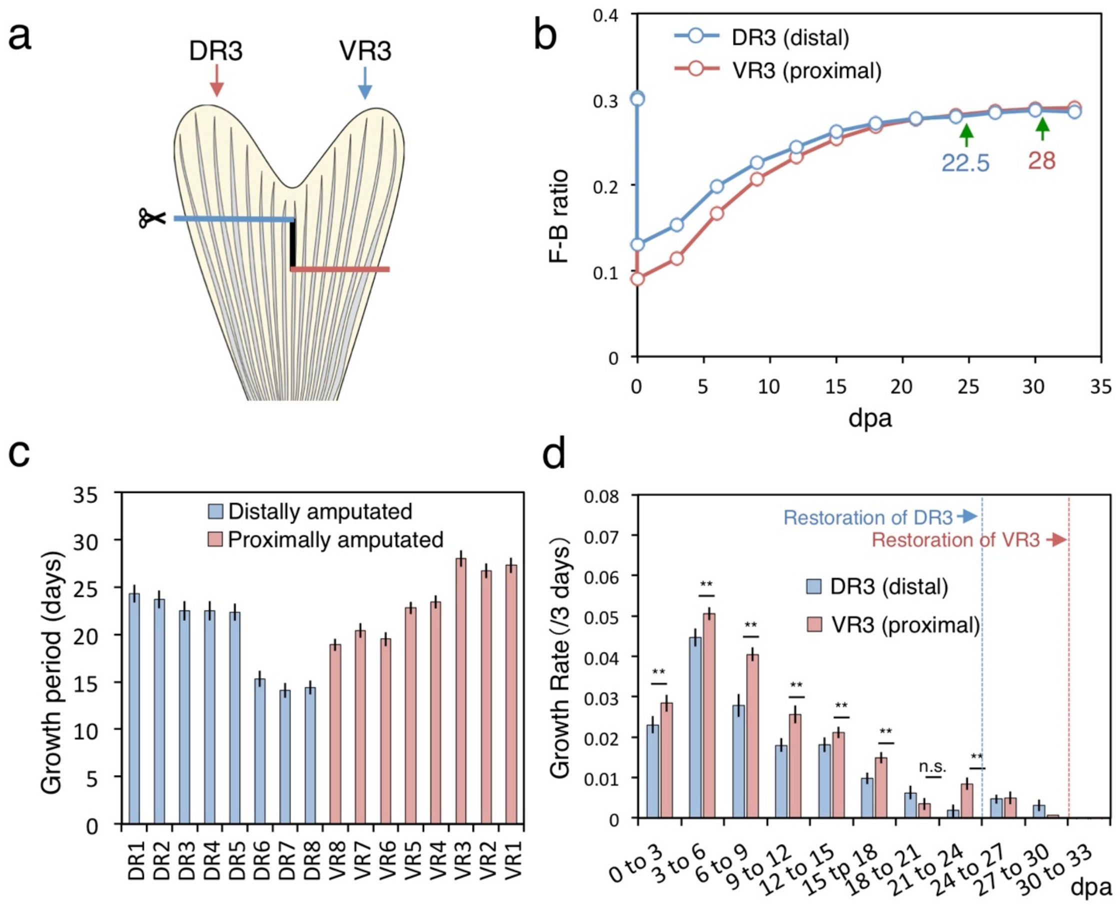

Supplementary figure 12. Growth analysis after stepwise (dorsal with distal, and ventral with proximal) amputation

(a) Schematic of the stepwise amputation. Dorsal fin rays (DR1-8) were amputated distally and ventral fin rays (VR1-8) were amputated proximally. (b) Transition of the F-B ratio for DR3 and VR3. Green arrows indicate the termination point of each fin ray. (c) Growth period of all fin rays. Blue and red bars indicate distally and proximally amputated fin rays, respectively. (d) Transition of the growth rate from 0 to 33 dpa in DR3 and VR3. Welch's t-test: p-value **< 0.01. n.s.: not significant. Error bars means ± SEM.

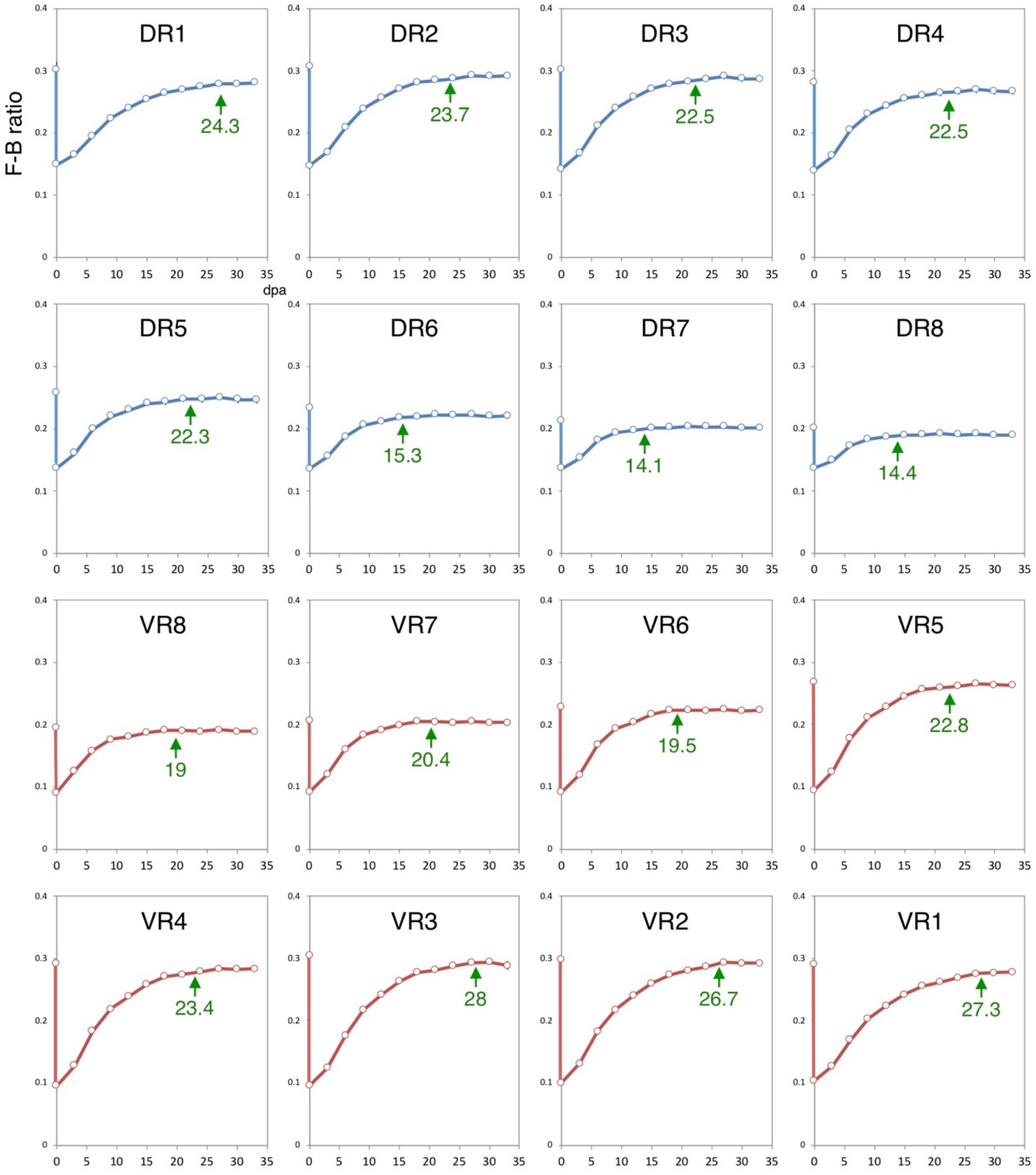

Supplementary figure 13. F-B ratio transition after stepwise (dorsal with distal, and ventral with proximal) amputation.

Transition of the F-B ratio for each fin ray. Green arrows indicate termination points. After termination, the F-B-ratios were almost constant. Blue and red lines indicate distally and proximally amputated fin rays, respectively. Error bars means ± SEM.

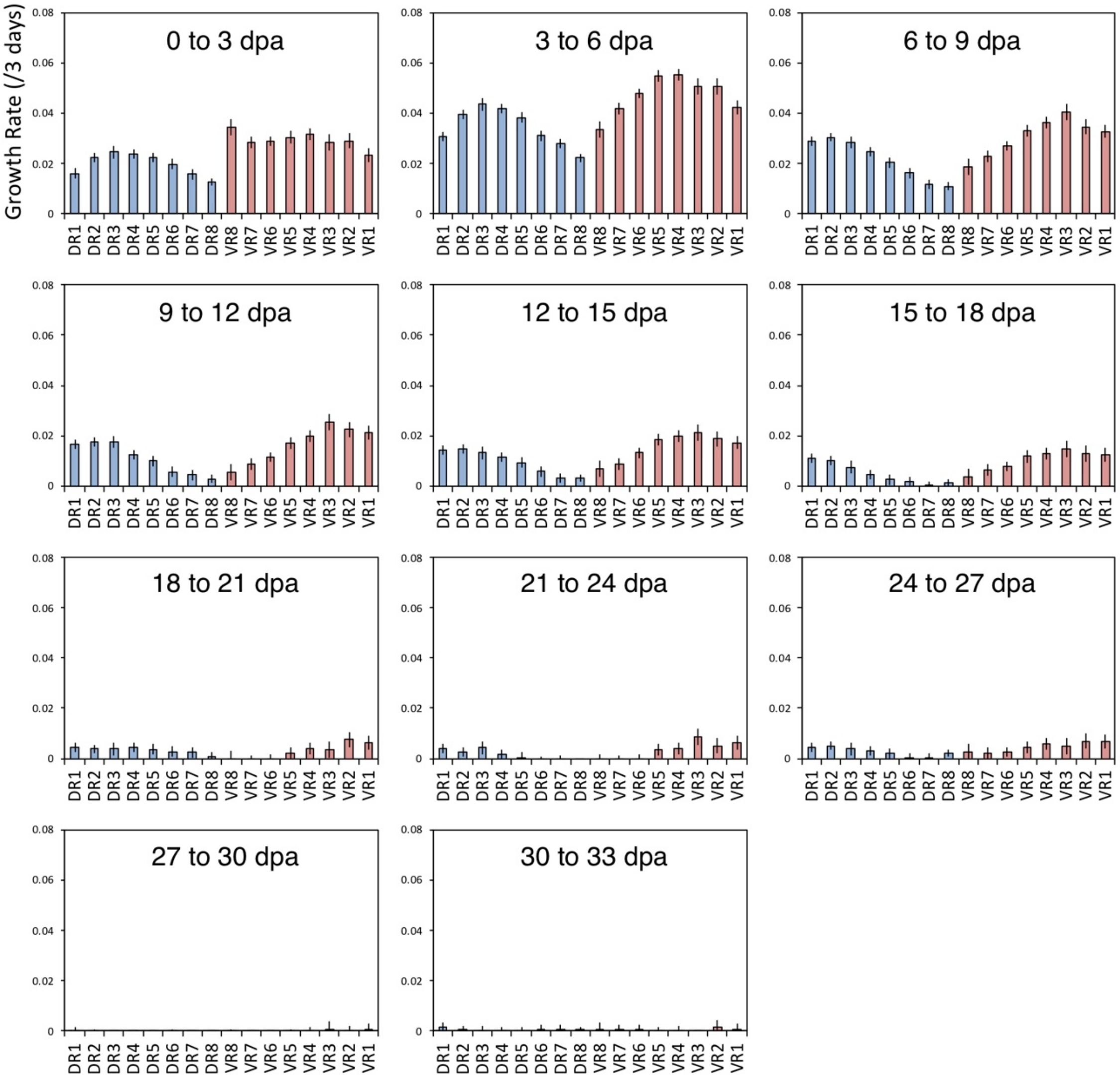

Supplementary figure 14. Growth rate of stepwise (dorsal with distal, and ventral with proximal) amputated fin rays during each growth period.

The growth rate from 0 to 33 dpa was calculated every three days. Blue and red bars indicate distally and proximally amputated fin rays, respectively. Error bars means ± SEM.

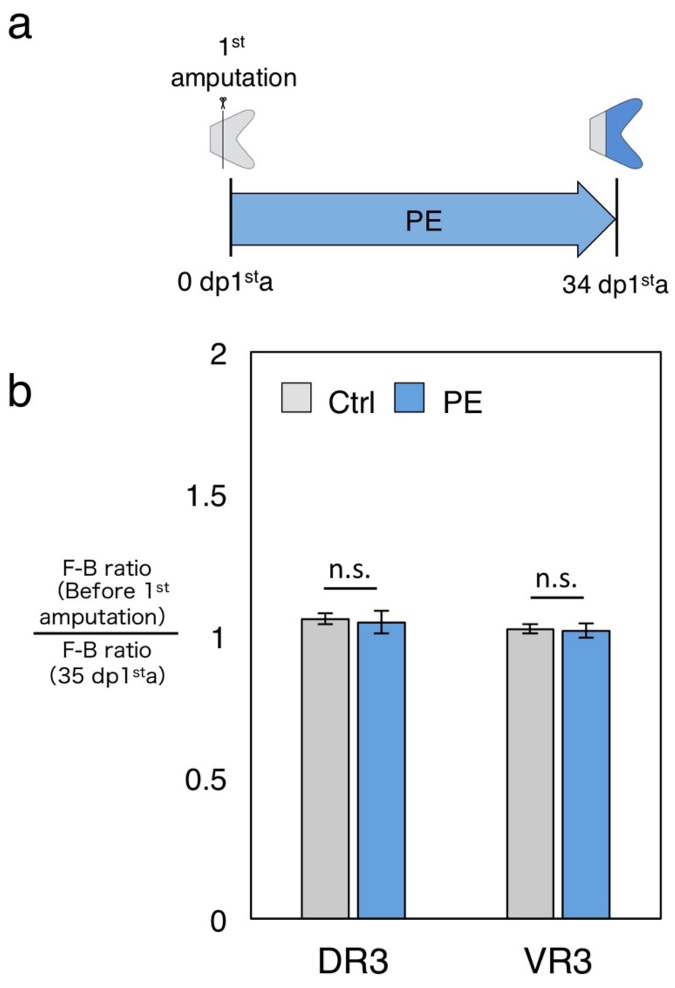

Supplementary Figure S15. Scheme of PE treatment without the second fin amputation.

(a) Scheme of the PE treatment. (b) F-B ratio of before the first amputation and on 34 dp1sta. dp1sta: days post first amputation. PE: phenylephrine. Welch's t-test: n.s.: not significant. Error bars indicate standard error of measurement.
